# Directed evolution of phosphite dehydrogenase to cycle noncanonical redox cofactors via universal growth selection platform

**DOI:** 10.1038/s41467-022-32727-w

**Published:** 2022-08-26

**Authors:** Linyue Zhang, Edward King, William B. Black, Christian M. Heckmann, Allison Wolder, Youtian Cui, Francis Nicklen, Justin B. Siegel, Ray Luo, Caroline E. Paul, Han Li

**Affiliations:** 1 Department of Chemical and Biomolecular Engineering, University of California Irvine, Irvine, CA 92697 USA; 2 Department of Molecular Biology and Biochemistry, University of California Irvine, Irvine, CA 92697 USA; 3 Biocatalysis, Department of Biotechnology, Delft University of Technology, 2629 HZ Delft, Netherlands; 4 Department of Chemistry, University of California, Davis, One Shields Avenue, Davis, CA 95616 USA; 5 Department of Biochemistry and Molecular Medicine, University of California, Davis, 2700 Stockton Boulevard, Suite 2102, Sacramento, CA 95817 USA; 6 Genome Center, University of California, Davis, 451 Health Sciences Drive, Davis, CA 95616 USA; 7 Department of Biomedical Engineering, University of California Irvine, Irvine, CA 92697 USA; 8 Department Materials Science and Engineering, University of California Irvine, Irvine, CA 92697 USA

**Keywords:** Applied microbiology, Enzymes, Enzyme mechanisms

## Abstract

Noncanonical redox cofactors are attractive low-cost alternatives to nicotinamide adenine dinucleotide (phosphate) (NAD(P)^+^) in biotransformation. However, engineering enzymes to utilize them is challenging. Here, we present a high-throughput directed evolution platform which couples cell growth to the in vivo cycling of a noncanonical cofactor, nicotinamide mononucleotide (NMN^+^). We achieve this by engineering the life-essential glutathione reductase in *Escherichia coli* to exclusively rely on the reduced NMN^+^ (NMNH). Using this system, we develop a phosphite dehydrogenase (PTDH) to cycle NMN^+^ with ~147-fold improved catalytic efficiency, which translates to an industrially viable total turnover number of ~45,000 in cell-free biotransformation without requiring high cofactor concentrations. Moreover, the PTDH variants also exhibit improved activity with another structurally deviant noncanonical cofactor, 1-benzylnicotinamide (BNA^+^), showcasing their broad applications. Structural modeling prediction reveals a general design principle where the mutations and the smaller, noncanonical cofactors together mimic the steric interactions of the larger, natural cofactors NAD(P)^+^.

## Abstract (web summary)

Engineering enzymes to utilize the noncanonical redox cofactors such as nicotinamide mononucleotide (NMN + ) is challenging. Here, the authors report a growth-based selection platform for NMN + -reducing enzyme engineering and show its application in developing a phosphite dehydrogenase with improved catalytic efficiency.

## Introduction

Enzymes catalyze many chemistries that are unattainable through organic synthesis. They perform these reactions renewably, operate under ambient conditions, and generate low waste^1,2^. The most prevalent applications of biocatalysis involves the regio- and stereo-selective synthesis of chemicals which is reliant on nicotinamide adenine dinucleotide (phosphate) (NAD(P)^+^)-dependent oxidoreductases^3,4^. These enzymes require stoichiometric input of NAD(P)^+^ for product formation, which constitutes a major cost that limits economic scalability^5–7^. Attempts to reduce input costs through cofactor regeneration pathways still do not decrease costs sufficiently^6,8^. This motivates the exploration of simpler NAD^+^ mimetics, or noncanonical redox cofactors, which retain the catalytic moiety of the native redox cofactors, but they are regularly structurally simpler and easier to synthesize^8–14^. However, a significant hurdle blocking the widespread use of these simpler noncanonical cofactors is the lack of efficient and diverse enzymes that can utilize them. With the exception of some flavoenzymes, most enzymes engineered to use noncanonical cofactors do so with catalytic activities too low for practical applications^15^.

Our previous work developed nicotinamide mononucleotide (NMN^+^), a truncated version of the native nicotinamide cofactors, as an efficient noncanonical redox cofactor^16,17^. Compared to other simpler NAD^+^ mimetics, NMN^+^ can be produced renewably using low-cost feedstocks through biosynthetic pathways^12,13,18–20^. More importantly, its polar structural features (particularly the phosphate) offer unique advantages for enzyme design. We previously demonstrated an engineered glucose dehydrogenase (GDH) that recycles NMN^+^ with the highest reported total turnover number (TTN), prior to this work, in noncanonical cofactor-based biotransformation^16^. In this report, we develop a facile, high-throughput, and universal growth selection platform to obtain NMN^+^-utilizing enzymes through directed evolution, enabling a practical application of the noncanonical redox cofactor NMN^+^ on demand.

**Fig. 1:**
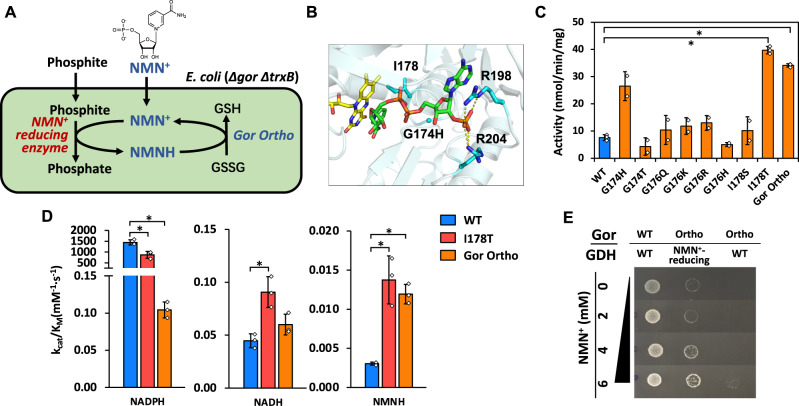
Development and validation of the Gor Ortho-mediated growth selection platform. **A** Schematic of NMN(H) redox system coupling NMN^+^ reducing enzyme and life-essential glutathione reductase in an engineered *E. coli* strain. Redox imbalance through low supply of NMNH results in insufficient GSH production and protein misfolding. **B** Structure of the coenzyme binding pocket in Gor from *E. coli* (PDB: 1GET). Residues interacting with the cofactors are shown as cyan sticks, the native cofactor NADPH is colored green, and the FAD is colored yellow. **C** In vitro NMNH-dependent activity of eight single-mutation variants in the first round of rational design. For WT, I178T, and Gor Ortho, *n* = 3 biologically independent replicates. For all other samples, *n* = 2 biologically independent replicates. For statistics, WT to I178T, *P* = 0.0001; WT to Gor Ortho, *P* = 0.000003. **D** Kinetic characterization of Gor variants. Activities were measured as described in Methods. *n* = 3 biologically independent samples. For statistics, NADPH WT to I178T, *P* = 0.007; NADPH WT to Gor Ortho, *P* = 0.002; NADH WT to I178T, *P* = 0.019; NMNH WT to I178T, *P* = 0.0038; NMNH WT to Gor Ortho, *P* = 0.0002. **E** Comparison of growth behavior for WT and *E. coli* SHuffle strain with coupled or uncoupled coenzyme recycling system at varying NMN^+^ concentrations. SHuffle with wild type Gor experiences no growth defects, while SHuffle with Gor Ortho only grows with NMNH regeneration. Error bars represent one standard deviation. Two-tailed *t*-tests were used to determine statistical significance (*P < 0.05). Source data are provided as a Source Data file.

Growth selection is a powerful tool in enzyme engineering due to its easy readout and unparallel throughput (>10^6^ per iteration)^15,21–28^, compared to 96-well plate-based or agar plate-based colorimetric methods (10^2^–10^4^ per iteration)^29–32^. Multiple growth-based selection platforms have been designed to engineer NAD(P)H-dependent enzymes^21–28^, where the unifying principle behind is that cells can only grow when the life-essential redox reactions have their cofactors recycled continuously in vivo. We extend this principle here for the noncanonical redox cofactor NMN^+^. The life-essential redox reaction we choose is the production of reduced glutathione (GSH) (Fig. 1A) in *E. coli*, which is required for cells to maintain their intracellular reducing environment and survive through oxidative stresses.

In this work, we first engineer this reaction to rely exclusively on NMNH by re-designing the cofactor specificity of glutathione reductase (Gor) (Fig. 1A). Next, we demonstrate selection of multiple efficient NMN^+^-recycling phosphite dehydrogenase^33^ (PTDH) variants from a site-saturated mutagenesis library, because only such variants can supply NMNH to power the engineered Gor (Fig. 1A). With our evolved PTDH variants featuring up to a ~110-fold increased catalytic efficiency toward NMN^+^ compared to the wild type enzyme, we achieve an industrially relevant TTN of ~45,000 with NMN^+^ in reduction biotransformation, the highest achieved so far for noncanonical cofactors. Furthermore, the PTDH’s low *K*_*M*_ value with NMN^+^ enable the process to operate at sub-millimolar cofactor concentrations. Interestingly, the engineered PTDHs also exhibit enhanced cycling of another even more cost-effective redox cofactor, 1-benzylnicotinamide (BNA^+^). Finally, we employ computational modeling to reveal a common archetype in which the mutations occupy the binding pocket for the adenosine monophosphate (AMP) motif which is present in NAD(P)^+^ but missing in NMN^+^ and other simpler NAD^+^ biomimetics. Overall, although PTDH is used as a proof-of-concept, we envision the selection platform will be used to evolve other noncanonical cofactor-dependent enzymes, because the redox balance-based mechanism is agnostic of the target enzyme’s substrate and product.

## Results

### Design of the growth-based selection platform for NMN^+^-reducing enzymes

GSH functions to reduce undesired cysteine disulfide bonds that impair protein folding, and GSH is required for the proper function of cytosolic proteins in *E. coli*. A parallel and partially redundant antioxidant system is the thioredoxin (Trx) system in *E. coli*. When both the GSH system and the Trx system are disrupted through the genetic knockout of Gor (glutathione reductase) and TrxB (thioredoxin reductase) in the *E. coli* SHuffle strain^34^, the intracellular environment becomes largely oxidative^35^. Although the SHuffle strain is still viable under non-stressful conditions, our hypothesis is that it cannot combat the oxidative stress effectively, and requires a functional Gor to survive exposure to oxidants such as diamide^36,37^. Thus, if Gor can be engineered to specifically utilize NMNH instead of the natural cofactors NAD(P)H, then virtually any NMN^+^-reducing enzyme can be made life-essential for *E. coli* by their role of supplying vital NMNH to the engineered Gor to support GSH production (Fig. 1A).

### Engineering an NMNH-specific Gor

NMN^+^ is a truncated version of the native cofactor NAD^+^ which maintains the nicotinamide ring that functions in hydride transfer and lacks the AMP recognition handle (Fig. 1A). We hypothesized that the ribose and phosphate groups of NMN(H) have the potential to accept new polar interactions to supplant the missing interactions from the AMP recognition handle. Our previous work has corroborated this hypothesis^16^.

The first round of rational design on *E. coli* Gor (Ec Gor) aimed to create additional interactions between the protein and the free phosphate group on NMNH. Three residue sites G174, G176, and I178 were targeted based on their proximity to the ribose-phosphate group of NMN(H), and a total of eight single-mutation variants were tested (Fig. 1B, C). The hydroxyl group on the side chain of T178 is predicted to make two new hydrogen bonds with the phosphate group of the NMNH ligand (Supplementary Fig. 1). Indeed, I178T had 4-fold enhanced NMNH-dependent activity compared to the wild type. Another variant predicted to make favorable salt bridge interactions with the NMNH phosphate group, G174H, also showed enhanced NMNH-dependent activity (Fig. 1B, C). These findings reinforced the design principle of introducing novel polar contacts to the NMNH phosphate group to increase activity.

We next sought to increase the orthogonality of Gor in using NMNH versus the natural cofactors NAD(P)H. The wild type Ec Gor strongly prefers NADPH, and it already has low binding affinity for NADH. Furthermore, a previously identified variant with R198M-R204L double mutations had almost completely abolished NADPH-dependent activity, possibly due to ablation of interactions with the 2′-phosphate group on NADPH^38^ (Fig. 1B). Combining the R198M-R204L double mutations on top of Ec Gor I178T caused only a modest decrease in NMNH-dependent activity (Fig. 1C, D). The catalytic efficiency (*k*_*cat*_/*K*_*M*_) for NMNH is comparable to that of the I178T, and is ~4-fold higher than the wild type. Importantly, the triple mutant Ec Gor I178T-R198M-R204L (named Gor Ortho) has drastically reduced catalytic efficiency for NADPH, by ~15,000-fold and ~9000-fold, respectively, compared to Ec Gor wild type and I178T single mutant (Fig. 1D). The additional R198M-R204L mutations also decreased the catalytic efficiency for NADH by ~2-fold (Fig. 1D), making Gor Ortho equally deficient in NADH activity as the wild type.

Gor Ortho features a ~60,000-fold cofactor specificity switch from its native cofactor NADPH to NMNH based on the catalytic efficiency measured in vitro (Fig. 1D). To assess its suitability for use in the growth selection in vivo, it is important to consider the intracellular concentrations of NADH and NADPH, ~83 µM and 120 µM, respectively, in glucose-fed exponentially growing cells^39^. During the kinetic characterization, Gor Ortho activity could not be saturated at concentrations up to 4 mM NAD(P)H, indicating large, non-physiologically relevant *K*_*M*_ for the natural cofactors (Supplementary Table 1). Although the catalytic efficiency for NMNH is still relatively low, the intracellular concentration of NMN(H) can be modulated by exogenously supplementing the cofactor in the growth media, which may support Gor Ortho’s NMN(H)-specific function in physiological conditions. We proceeded to test Gor Ortho in the SHuffle strain for NMN(H) cycling-dependent growth behavior.

### Validation of the growth-based selection platform

The functional selection platform must specifically report the presence of an NMN^+^-reducing enzyme using growth as a readout. Our previous work established an engineered GDH, *Bacillus subtilis* GDH Triple (I195R-A93K-Y39Q)^16^, which can efficiently reduce NMN^+^ using glucose. When this engineered Bs GDH Triple mutant is introduced to the SHuffle strain which also harbors Gor Ortho, cell growth was observed and found to increase with greater NMN^+^ supplementation in the media (Fig. 1E). The selection media contains 0.5 mM diamide which has been optimized to exert the appropriate level of oxidative stress. Importantly, wild type Bs GDH, which only reduces NAD(P)^+^ but not NMN^+^, did not support cell growth (Fig. 1E). In comparison, the SHuffle strain harboring wild type Ec Gor exhibited robust growth independent of NMN^+^ supplementation since wild type Ec Gor can rescue growth by producing GSH using intracellular NADPH (Fig. 1E). These results demonstrate that the growth selection platform functions as intended, with the SHuffle strain carrying Gor Ortho supporting growth only with NMN^+^ cycling.

### Enable phosphite dehydrogenase to use NMN^+^ by rational design

PTDH has been widely applied for cofactor regeneration in industrial biocatalytic processes due to its usage of the low-cost feedstock phosphite (Pt), production of the non-toxic product phosphate (Pi) that has additional pH buffering ability, and reaction irreversibility that can be exploited as a thermodynamic driving force^33^.

To engineer an NMN^+^-recycling PTDH, we started with the 16X thermostable variant of *Pseudomonas stutzeri* PTDH (TS-PTDH)^40^ and employed a two-stage approach: First, rational design was carried out on residue positions that directly contact NMN^+^; Second, site-saturated mutagenesis was performed targeting sites that are more remote to NMN^+^ in the cofactor binding pocket combined with high-throughput screening by the Gor Ortho growth selection platform. This two-stage workflow integrating structure-based design and semi-rational mutagenesis enables the discovery of NMN^+^ active mutants with minimized experimental burden. The first round of design is performed to quickly acquire rationally apparent mutations that can seed exploration in the second round. Subsequently, resources are concentrated on the second round sampling with high combinatorial variation to avoid getting trapped in local optima that pervade protein fitness landscapes^41^.

**Fig. 2:**
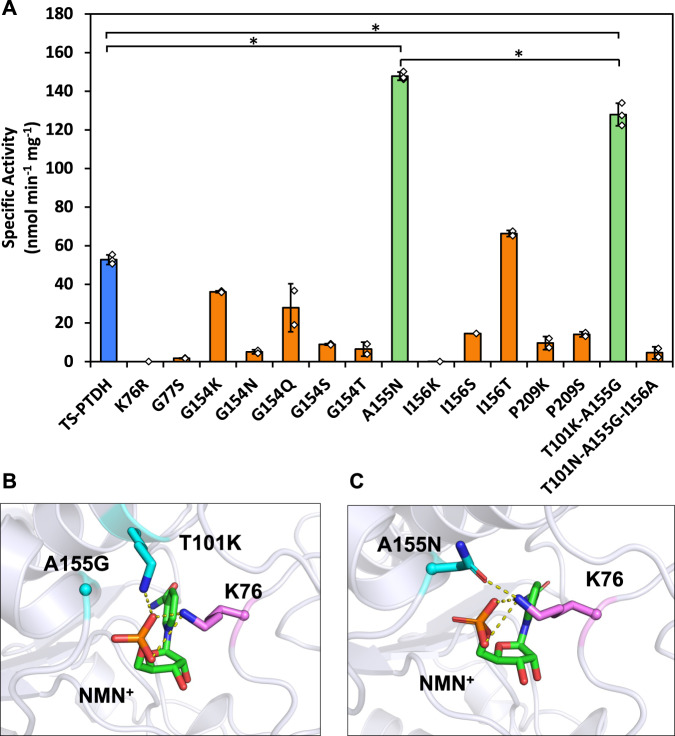
First round of TS-PTDH variants engineered through rational design. **A** Two mutants (green) displayed specific activities with NMN^+^ notably greater than the wild type (blue). A155N showed ~3-fold increase and T101K-A155G showed a ~2-fold increase compared to the wild type. For TS-PTDH, A155N, and T101K-A155G, *n* = 3 biologically independent replicates. For other candidates, *n* = 2 biologically independent replicates. For statistics, TS-PTDH to A155N, *P* = 9 × 10^−7^; TS-PTDH to T101K-A155G, *P* = 0.0004; A155N to T101K-A155G, *P* = 0.018. **B** Molecular modeling of the designs suggest that T101K-A155G functions through the cooperative effects of A155G clearing space for T101K to project over to form a salt bridge with the NMN^+^ phosphate. **C** A155N forms a hydrogen bond with K76 that reinforces the existing salt bridge between K76 and the phosphate group. This interaction may support pre-organization of K76 to grasp the cofactor upon binding. Values represent the average of at least two biological replicates. Error bars represent one standard deviation. Two-tailed *t*-tests were used to determine statistical significance (*P < 0.05). Source data are provided as a Source Data file.

For the initial round of rational design, we sought to mutate first shell residues to form novel polar contacts to NMN^+^, in particular at the phosphate or ribose (Supplementary Figs. 2 and 3). Evolutionary conservation for regions surrounding the NMN^+^ binding pocket was calculated through sequence entropy of aligned homologs to identify sites with high variability or those prone to sample polar residues (Supplementary Figs. 2 and 3; Supplementary Method 1), which show that all NMN^+^ first shell residues are highly conserved. However, studies have shown that caution should be taken in equating sequence consensus to functional essentiality^42^, especially when the desired function (i.e., NMN^+^-utilizing activity) has not been explored by evolution. Instead of bioinformatic analysis, we next relied on a structure-guided approach (Supplementary Fig. 4) and designed 14 variants (Fig. 2A) to enhance NMN^+^ activity. Of the 11 residues lining the NAD binding pocket, 6 residues were selected for rational design after eliminating residues distal to the NMN phosphate and residues critical for catalytic function. These 6 residues were mutated to polar amino acids which were hypothesized to be able to form novel polar contacts with NMN^+^.

Through specific activity assay we identified two variants of interest displaying enhanced NMN^+^-dependent activity, A155N and T101K-A155G. The template TS-PTDH displayed NMN^+^-dependent specific activity of 53.0 ± 3.4 nmol min^−1^ mg^−1^, while T101K-A155G showed roughly twofold, and A155N around threefold increase (Fig. 2A). Further kinetic analysis of A155N indicated a ~3-fold increase in catalytic efficiency for NMN^+^ compared to TS-PTDH (Table 1).

**Table 1:** Kinetic parameters of PTDH variants

Enzyme	Kinetic parameters								
	NAD^+^			NADP^+^			NMN^+^		
	*k*_*cat*_ [s^−1^]	*K*_*M*_ [mM]	*k*_*cat*_/*K*_*M*_ [mM^−1^ s^−1^]	*k*_*cat*_ [s^−1^]	*K*_*M*_ [mM]	*k*_*cat*_ /*K*_*M*_ [mM^−1^ s^−1^]	*k*_*cat*_ [s^−1^]	*K*_*M*_ [mM]	*k*_*cat*_ /*K*_*M*_ [mM^−1^ s^−1^]
TS-PTDH	1.03 ± 0.01	0.06 ± 0.01	18 ± 1	0.57 ± 0.01	0.74 ± 0.04	0.77 ± 0.03	n.d.^a^	n.d.^a^	0.004 ± 0.001
TS-PTDH A155N	1.46 ± 0.13	0.08 ± 0.01	18 ± 1	0.88 ± 0.08	0.63 ± 0.07	1.4 ± 0.1	n.d.^a^	n.d.^a^	0.010 ± 0.001
TS-PTDH A155N-E175Q-A176S (LY-6)	1.04 ± 0.08	0.11 ± 0.01	9.3 ± 0.4	0.95 ± 0.05	0.05 ± 0.01	19 ± 1	0.18 ± 0.01	1.1 ± 0.1	0.17 ± 0.01
TS-PTDH A155N-E175W-A176G-L208V (LY-7)	0.71 ± 0.04	0.75 ± 0.03	0.94 ± 0.08	0.56 ± 0.03	0.11 ± 0.01	5.2 ± 0.2	0.16 ± 0.01	1.4 ± 0.1	0.11 ± 0.01
TS-PTDH A155N-E175A-A176F (LY-13)	2.06 ± 0.11	0.18 ± 0.03	12 ± 2	0.96 ± 0.02	0.05 ± 0.01	20 ± 1	0.27 ± 0.01	0.62 ± 0.05	0.44 ± 0.03

Reactions were performed in 100 mM MOPS, pH 7.25, 10 mM sodium phosphite, and varied cofactor concentrations. Values reported with standard deviations and replicate size of *n* = 3. Source data are provided as a Source Data file.

*K*_*M*_ values are higher than 5 mM as the enzyme could not be saturated with the cofactor concentrations tested. For *k*_*cat*_/*K*_*M*_, the Michaelis–Menten equation was modified under the assumption *K*_*M*_ » S^16^. Using the modified equation, we performed linear regression on initial reaction rate versus NMN^+^ concentration, the *k*_*cat*_/*K*_*M*_ was determined using the resulting slope divided by enzyme concentration.

^a^Not detectable.

To determine the structural basis for the improved catalysis with NMN^+^, we performed molecular modeling with Rosetta to examine potential binding modes. T101K reaches across the scaffold of the cofactor to form a salt bridge with the NMN^+^ phosphate, and A155G was necessary to clear space for T101K (Fig. 2B). A155N extends over the NMN^+^ phosphate and forms a hydrogen bond with K76, reinforcing the existing salt bridge between K76 and the NMN^+^ phosphate^43^ (Fig. 2C). This interaction is suggested to pre-organize K76 for cofactor binding. We continued engineering the variant with the highest NMN^+^ activity, A155N.

### Evolving phosphite dehydrogenase using high-throughput growth selection

We next constructed a site-saturated mutagenesis library on top of TS-PTDH A155N targeting E175, A176, and L208. These three positions span the binding cleft surrounding the adenosine moiety of NAD^+^ and are part of the key determinants of cofactor specificity in TS-PTDH^43,44^. These three sites were mutated simultaneously using NNK degenerate codons, and the resultant library’s quality was determined as described by Stewart and coworkers^45^ (Supplementary Fig. 5). The library was transformed into the SHuffle strain together with Gor Ortho to produce ~2.5 × 10^6^ independent transformants, ensuring thorough coverage of the theoretical diversity (8 × 10^3^ variants) with fivefold oversampling.

In the first round of selection carried out on M9 agar plates with 0.5 mM diamide, 10 g/L glucose, 10 g/L sodium Pt, 5 g/L yeast extract, and 5 mM NMN^+^, hundreds of transformants grew as isolated colonies after incubation at 37 °C for 3 days. Eight colonies were picked due to their large colony size and robust growth when re-streaked on plates with identical medium. From the 8 colonies, four PTDH variants were isolated that showed enhanced NMN^+^-dependent activity compared to the template TS-PTDH A155N (data not shown). Among them, TS-PTDH A155N-E175Q-A176S (LY-6) and TS-PTDH A155N-E175W-A176G-L208V (LY-7) exhibited the greatest improvement, with ~17-fold and ~11-fold increased catalytic efficiency (*k*_*cat*_/*K*_*M*_) for NMN^+^ compared to the parent enzyme TS-PTDH A155N, respectively (Table 1).

In the second round of selection, we increased the selection pressure by lowering the yeast extract concentration to 1 g/L, making the condition even more challenging for cells to grow. Using the same growth selection process, we successfully isolated TS-PTDH A155N-E175A-A176F (LY-13) which has a catalytic efficiency for NMN^+^ ~110-fold and ~44-fold higher than that of TS-PTDH and TS-PTDH A155N, respectively, reaching 0.44 mM^−1^ s^−1^ (Table 1). When retransformed into the growth selection strain, LY-13 demonstrated a markedly improved growth restoration relative to the A155N and wild type TS-PTDH (Supplementary Fig. 6). Intriguingly, these improvements in TS-PTDH activity with NMN^+^ are also shown with NADP^+^, with only a minimal decrease in activity with the preferred NAD^+^ in most cases, indicating these mutations may contribute to a global improvement in activity with a broad range of nicotinamide-based redox cofactors (Table 1).

### Elucidating the mechanism of enhanced NMN^+^-dependent activity in engineered PTDHs

**Fig. 3:**
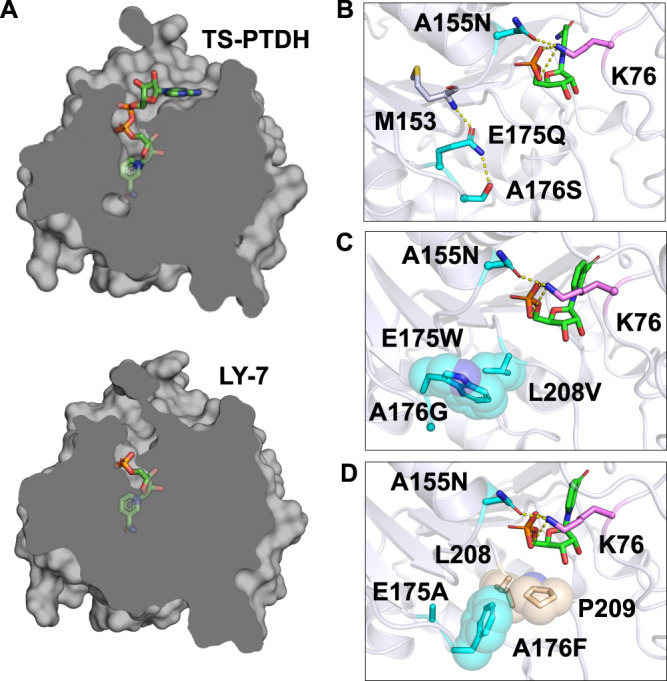
Structural modeling of PTDH variants discovered through growth selection with A155N template. **A** Cartoon of TS-PTDH depicting residue packing at the AMP binding pocket in LY-7. In TS-PTDH, NAD^+^ is docked in the binding pocket. In LY-7, NMN^+^ is docked in the binding pocket. **B** In LY-6 (A155N-E175Q-A176S), E175Q maintains similar steric properties of the original glutamate, but the change from the carboxylic acid to amide enables E175Q to act as a hydrogen bond donor that bridges separate loops in the adenine binding cleft for improved stability. The backbone amide of M153 contacts the oxygen on E175Q and the hydroxyl from A176S accepts a hydrogen bond from the E175Q nitrogen. **C** LY-7 (A155N-E175W-A176G-L208V) is characterized by dense hydrophobic packing in the adenine cleft. A176G allows greater loop flexibility and room for E175W to adopt a rotamer that occupies the open void where the adenine would typically fit. L208V results in a slightly smaller side chain with greater shape complementarity to pack against the bulky E175W. **D** LY-13 (A155N-E175A-A176F) displays similar hydrophobic packing in the adenine cleft as LY-7 with E175A shrinking and A176F settling against L208 and P209 in the adenine cleft.

The variants identified from growth selection were further analyzed through Rosetta modeling to understand the mechanisms leading to enhanced NMN^+^-dependent activity. A common pattern observed is additional residue packing in the adenine cleft, which potentially recapitulates the role of the missing AMP when NMN^+^ is bound (Fig. 3A).

LY-6 (TS-PTDH A155N-E175Q-A176S) is predicted to support the extension of E175Q into the space where the adenine would occupy. E175Q is able to form two hydrogen bonds, one with the backbone amide of M153 and the second with the side chain hydroxyl of A176S. These hydrogen bonds stabilize the positioning of E175Q into the binding pocket and the neighboring loops (Fig. 3B). Both LY-7 (A155N-E175W-A176G-L208V) and LY-13 (A155N-E175A-A176F) display tight hydrophobic packing that bridges the gap between the loop emerging from the second Rossman beta strand and the loop below the bound cofactor. In LY-7, the substitution of A176G allows greater rotamer sampling for E175W, and E175W is able to twist and face L208V. L208V results in a slightly smaller residue that maintains non-polar character and packs with high shape complementarity against E175W to fill the adenine pocket (Fig. 3C). In LY-13, the smaller E175A allows the specificity loop to move closer toward the GXGXXG glycine rich signature loop in the Rossman fold. The bulky A176F pads the void where the adenosine would fit, and it achieves minimal exposure to solvent by lying against L208 and P209 (Fig. 3D).

### Application of the engineered PTDHs in biocatalysis using noncanonical cofactors

**Fig. 4:**
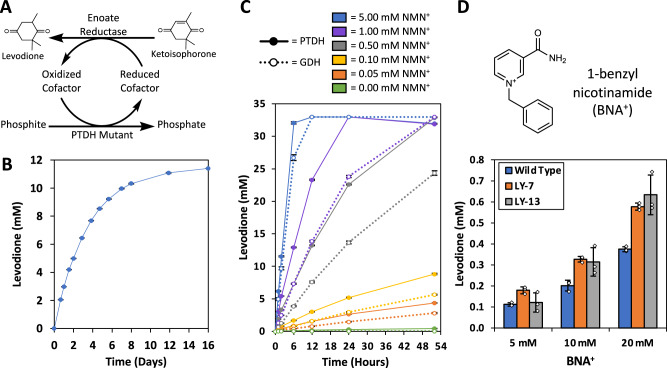
Applying TS-PTDH to reductive biotransformation. **A** A redox cofactor cycling system was constructed using PTDH as a reduced cofactor regeneration source. **B** Reaction progression curve for determining the TTN of LY-13 in the redox cycling system employing NMN(H). A low concentration of LY-13, 0.25 µM, requires high protein turnover to enable the reaction to progress. **C** Comparison of using LY-13 or an NMNH-active glucose dehydrogenase (GDH Triple) as NMNH recycling enzymes. 10 µM LY-13 or GDH Triple were supplied to the cycling system containing varying concentrations of NMN^+^. The lower *K*_*M*_ of LY-13 enables more efficient utilization of the NMN^+^ pool at lower cofactor concentrations. **D** The synthetic noncanonical cofactor BNA^+^ was substituted for NMN^+^ in the cycling system. Despite the highly deviant structure of BNA^+^ compared with NMN^+^, the TS-PTDH variants were able to recycle BNA(H) at a higher rate than the wild type protein. Values represent the average of three biological replicates. Error bars represent one standard deviation. Source data are provided as a Source Data file.

To examine the application of the TS-PTDH variants as noncanonical redox cofactor recycling tools, we constructed an in vitro enzymatic cycling system (Fig. 4A), coupling the TS-PTDH variants with the xenobiotic ene reductase XenA from *Pseudomonas putida*. This forms a closed NMN(H) cycle producing the flavor and fragrance compound levodione from ketoisophorone (KIP). First, we sought to investigate the temporal stability of LY-13. LY-13 was supplied at a low concentration of 0.25 µM to the reaction system, requiring numerous turnovers of LY-13 for the reaction to progress (Fig. 4B). This PTDH-limited reaction system remained active for the entire 16 days it was observed, achieving a TTN of ~45,000 with LY-13 (Fig. 4B), a Total Turnover Frequency of ~117 turnovers per hour averaged over the 16 day reaction. This represents the highest TTN value reported to date for a noncanonical cofactor cycling system^16,46–49^, and it approaches the TTN values reported for the wild-type PTDH enzyme with NAD^+^ (80,000)^50^. In addition to its robust temporal stability, the low *K*_*M*_ of LY-13 with NMN^+^ (Table 1) has been shown to be advantageous (Fig. 4C). When the cycling system utilizing LY-13 was directly compared with a system using GDH Triple, a previously reported NMN^+^ recycling enzyme (*K*_*M*_ = 6.4 mM, *k*_*cat*_ = 3.1 s^−1^)^16^, both systems completely converted the 33 mM KIP in 12 h when 5 mM of NMN^+^ was supplied (Fig. 4C). However, when the NMN^+^ supplementation was decreased to 1 mM and lower, the LY-13 enabled higher biotransformation rates and final titers compared to GDH Triple (Fig. 4C).

With the perspective of industrial scale, the cost of cofactor can be further decreased by switching NMN^+^ for a more cost-effective cofactor. We further explored the cofactor versatility of the PTDH variants, by exchanging NMN^+^ for an inexpensive, synthetic cofactor, BNA^+^ in the biotransformation system^51^. In addition, XenA was exchanged with another ene reductase from *Thermus scotoductus*, *Ts*OYE, which has been reported to exhibit improved activity with the reduced BNA^+^ (BNAH)^51^. When BNA^+^ was reduced with wild type TS-PTDH, LY-7, and LY-13, a cofactor dose-dependent response in biotransformation efficiency was observed, and at 20 mM BNA^+^ supplementation, LY-7 and LY-13 showed improved utilization of the BNA^+^ relative to the wild type protein (Fig. 4D). Interestingly, the relative increase in BNA(H)-dependent biotransformation efficiency was consistent with the improvements in NMN^+^ catalytic efficiency (Table 1), regardless of, albeit much lower in absolute terms due to the drastic structural differences between these two noncanonical cofactors (Fig. 4D). This supports the hypothesis that the adenine-cleft filling mutations, regardless of the NMN^+^-hydrogen binding mutation (Supplementary Fig. 7), are responsible for more global changes in the protein which enhanced acceptance of noncanonical cofactors in general.

## Discussion

The impact of this work is described by three contributions. First, the Gor-based growth selection platform can be used to rapidly develop NMN^+^-utilizing enzymes, which may enable more economical and scalable biotransformation. Second, the PTDHs developed here are highly proficient catalysts in recycling noncanonical redox cofactors due to their superior TTN, ability to maintain turnover at low NMN^+^ concentrations, and capability to recycle other simpler cofactor biomimetics. Third, through deep searching of protein sequence space enabled by our high-throughput selection, a general design principle of establishing binding interactions that emulate the effects of having the native cofactor bound began to emerge which may shed light on the engineering of other noncanonical redox cofactor-dependent enzymes.

Compared to previously reported colorimetric assay-based approaches^29–32^, utilizing growth as a simple readout afforded higher throughput which enabled us to observe a strong trend of convergent evolution: multiple NMN^+^-reducing variants employ very different sets of mutations to achieve the same predicted mechanism (recapitulating AMP) to enhance the NMN^+^-dependent activity. In particular, LY-6 utilized a hydrogen bond network to brace the AMP binding pocket, which is in stark contrast with the hydrophobic packing mode utilized by LY-7 and LY-13 (Fig. 3). In addition, growth-based selection can be augmented with in vivo, continuous mutagenesis and adaptive laboratory evolution to more closely mimic the depth and scale of natural evolution that Nature explores in navigating protein sequence space to yield enzymes with natural-like levels of activity^52–57^. The high-throughput nature of this tool is particularly advantageous in shaping complex enzyme behavior, such as modulating conformational or allosteric dynamics^58^.

The main bottleneck in developing growth-based selections is that cell metabolism must be tailored case-by-case to make the desired enzymatic activity essential for cell survival. This task is especially challenging when engineering enzymes for biotechnology applications, as most of the industrially important reactions neither exist in natural metabolism nor do they contribute to cell fitness. However, employing the redox balance principle can bypass this bottleneck in engineering redox cofactor-dependent enzyme^15,21–23,25,59^. In this work, we established the engineered Gor as a universal reporter for intracellular availability of NMNH, which successfully distinguished the high and low NMN^+^-reducing variants of two distinct enzymes, PTDH and GDH. This selection platform is readily adaptable to engineering other enzymes. If Gor is engineered to accept other noncanonical cofactors such as BNA^+^, the scope of selection can be further broadened.

The AMP moiety of NAD(P)^+^ does not participate in redox reactions, but studies suggest that it may influence the enzymatic activity by modulating protein conformational dynamics^60,61^. Indeed, crystallography studies on TS-PTDH reveal that the cofactor binding shifts the enzyme into a closed conformation, with a more compact active site and a better positioned K76 side chain for favorable interaction with the NMN^+^ phosphate^43^. Therefore, it is possible that similar effects can be induced by the AMP-recapitulating mutations in the engineered PTDHs, which would be the basis for both the lowered *K*_*M*_ toward NMN^+^ and the enhanced *k*_*cat*_. Structural studies and molecular dynamics simulations may be used to investigate this hypothesis. Interestingly, these AMP-recapitulating mutations also benefit BNA^+^ utilization. Therefore, this design principle may be added to the toolbox of rationally engineering BNA^+^-utilizing enzymes, in addition to the more obvious approach to directly contacting the benzyl recognition handle with strengthened van der Waals interactions.

Ultimately, the adoption of noncanonical redox cofactors in industrial processes is largely reliant on the availability of proteins capable of regenerating spent cofactor which are stable, utilize inexpensive substrates or waste streams, and catalytically active over a broad spectrum of reaction conditions. Ideally, these proteins exhibit low *K*_*M*_ and high *k*_*cat*_ values, enabling a highly productive system which operates at maximum efficiency over the course of their lifetime. However, fine tuning these specific parameters has largely proven difficult, especially when engineering for novel activity with noncanonical cofactors^46,49^. The tunable, iterable, and inter-generational nature of this growth selection platform is uniquely suited to select for enzymes with improved catalytic efficiencies.

## Methods

### Media and growth conditions

Bacterial strains and plasmids used in this work are described in the Supplementary Data 1. Relevant accession codes used in this work are described in the Supplementary Data 2. The wild-type *Escherichia coli* strain NEB express T7 and its derivative mutants SHuffle T7 Express (ΔtrxB, Δgor, ahpC* + cytoplasmic DsbC), were used for growth-based selection. XL-1 Blue was used to propagate all plasmids. BW25113 *Δgor::kan* obtained from the Yale *E. coli* Genetic Stock Center, was used to express Gor variants. BL21(DE3) was used to express all the other proteins. *E. coli* cells were cultured in 2xYT media containing 16 g/L tryptone, 10 g/L yeast extract, 5 g/L NaCl and appropriate antibiotics. M9 media contains 1 mM MgSO_4_, 0.1 mM CaCl_2_, trace metal mix A5 with Co (H_3_BO_3_ 2860 µg/L H_3_BO_3_ 2860 µg/L, MnCl_2_ · 4H_2_O 1810 µg/L, ZnSO_4_ 7H_2_O 222 µg/L, Na_2_MoO_4_, 2H_2_O 390 µg/L, CuSO_4_, 5H_2_O 79 µg/L, Co(NO_3_)_2_·6H_2_O (49 µg/L), and BD Difco M9 salts (Na_2_HPO_4_ 6.78 g/L, KH_2_PO_4_ 3 g/L, NaCl 0.5 g/L, NH_4_Cl 1 g/L). Concentrations utilized for antibiotic selection were 100 mg/L for ampicillin, 50 mg/L for spectinomycin, 50 mg/L for kanamycin, and 10 mg/L for chloramphenicol. Induction was initiated with final concentrations of 0.5% arabinose for strains with *P*_*BAD*_ promoter, and 0.5 mM Isopropyl-ß-D-thiogalactopyranoside (IPTG) for strains with *P*_*LlacO1*_ promoter. All strains were cultured at 37 °C with 250 r.p.m. agitation unless otherwise noted. Cell growth and enzyme assay were collected using a SpectraMax plate reader with SoftMax Pro 7.0 software.

### Plasmid and TS-PTDH library construction

The *E. coli* Gor gene was amplified from *E. coli* BW25113 chromosomal DNA. The TS-PTDH was amplified from a synthesized DNA template (Integrated DNA Technologies, San Diego, CA). PCR fragments were generated using PrimeSTAR Max DNA Polymerase (TaKaRa) unless otherwise noted. After PCR and gel extraction, gene fragments were assembled with vector backbones (pQElac gap Amp^R^, pRSF ori, Kan^R^, or pQE Amp^R^) using Gibson isothermal DNA assembly method^62^, resulting in plasmids pLZ 301, 311, 313 and pEK 201. Plasmids carrying the Gor or PTDH mutations were generated using the corresponding wild type plasmids as a template and using site-directed mutagenesis to introduce single or multiple mutations.

The PTDH combinatorial site-saturation mutagenesis library pLZ316 was constructed with degenerate codon-containing primers. Briefly, PCR was used to amplify a DNA fragment from pLZ314 using a forward primer containing degenerate codons NNK at E175 and A176 positions together with a reverse primer containing degenerate codons MNN at L208. These two fragments were used as templates in a splicing-by-overlap extension PCR. The resulting fragment was assembled with a complementary fragment that was generated by PCR of the same template to amplify the rest of pLZ314 by Gibson assembly^62^. The assembled plasmid was transformed into ElectroMAX DH10B cells (Invitrogen) by electroporation. Subsequently, the cells were rescued with SOC media at 37 °C for 1 h with shaking and added into 20 mL 2xYT media with ampicillin. 0.2, 1 and 5 μL of culture were immediately taken from the culture and plated on an 2xYT agar plates with ampicillin. The plate was incubated at 37 °C overnight, and the number of colonies formed was counted to estimate the library size. The liquid culture was incubated at 37 °C with shaking for 10 h, the library DNA was extracted using QIAprep Spin Miniprep Kit (Qiagen). Six single colonies from the library estimation plates were cultured individually to extract plasmids, which were sequenced as representatives of the population. The results showed that all six plasmids contained unique mutation patterns, and no other mutations outside the intended mutagenesis sites were observed. The library size of pLZ316 was counted as 4.7 × 10^7^ transformants.

### Expression and purification of Gor wild-type and variants

*E. coli* Gor (EC 1.6.4.2) is a homo-dimeric flavoenzyme containing 450 amino acid residues with 1 FAD per subunit. To prevent interference from endogenous protein, Gor wild-type and variants were expressed using *E. coli Δgor::kan* strain containing pQElac based plasmids encoding for the Gor wild-type or variants. 1 % (v/v) cells from an overnight culture were cultured in 2xYT media with 200 μg/mL ampicillin and 50 μg/mL kanamycin in shake flasks at 37 °C in a rotary shaker to an OD_600nm_ of 0.4–0.6. The cultures were then induced with 0.5 mM IPTG and incubated at 30 °C with shaking for 24 h. The cells were harvested with centrifugation and lysed by bead beating with Zymo His Binding Buffer (Zymo Research, CA, USA). Protein purification was performed with Zymo His-Spin Protein miniprep purification kit according to the manufacturer’s instructions. The concentrations of purified protein were quantified by Coomassie dye–based assays (Bradford) using BSA (Bovine Serum Albumin) as standards.

### Specific activity and steady state kinetic analyses for Gor wild-type and variants

The specific activity and kinetic parameters of Gor with the different cofactors, shown in Fig. 1, were measured using the following methods^63^. Briefly, for measuring the specific activity with NMNH, the 100 µL reaction mixture contained 96 mM potassium phosphate buffer pH 7.5, 2.5 mM GSSG and 0.8 mM NMNH. NMNH was generated using the following methods^16^. Briefly, 4 mM NMN^+^ was incubated in a solution containing 50 mM Tris-Cl at pH 8.5, 0.2 mM EDTA, 50 mM Na2HPO4, 5 mM D-glyceraldehyde-3-phosphate, and 28 µM of purified *E. coli* glyceraldehyde-3-phosphate dehydrogenase. The reaction was incubated at 30 °C for 8 h. The protein was removed by an Amicon Ultra 3 K filter, and the flow-through was dried under vacuum. Assays investigating the steady-state kinetic parameters were completed with 100 μL reaction mixtures containing 96 mM Tris-Cl buffer pH 7.5, 2.5 mM GSSG and varied cofactor concentrations from 0.01 mM to 1.6 mM. The assay was started by addition of 10 μL of purified protein at appropriate concentration at 25 °C. The kinetic parameters were measured by monitoring the NAD(P)H consumption at 340 nm in 96-well plate. Data were fit to the Michaelis–Menten equation to generate estimates of apparent catalytic efficiency (*k*_*cat*_/*K*_*M*_). The results are the average of three independent experiments.

### Culture conditions for GDH-Gor coupled growth rescue in SHuffle strain

The electro-competent cells of SHuffle harboring pLZ311 or pLZ312 were made as follows: The SHuffle cells were cultured in 200 mL SOB media with spectinomycin at 30 °C with shaking until OD_600nm_ reached 0.4–0.6. The culture was chilled on ice for 15 min and the cells were pelleted at 4 °C, 4000 × g. Collected cells were washed with 10% glycerol in water (sterile, ice cold) and resuspended with 5 mL of 10% glycerol in water (sterile, ice cold), and stored as 50 µL aliquots at −80 °C.

Glucose facilitator (encoded by *glf*) was used for uptake of unphosphorylated glucose into the cells^16^. The co-transformation of *Bs gdh* and *Zm glf* was performed as follows: Thaw SHuffle/pLZ311 or SHuffle/pLZ312 electro-competent cells on ice, add 1 μL *Bs gdh* (pEK101 or pLZ210) and 1 μL *Zm glf* (pSM109) plasmid DNA to 50 µL competent cells, mix, and electroporate. Cells were recovered with 1 mL SOC at 37 °C with shaking for 1 h. The cells were added to 10 mL 2xYT with 10 g/L glucose, 0.5% arabinose, kanamycin, chloramphenicol, and ampicillin. After incubation at 37 °C with shaking for 10 h, 1 mL cells were washed three times and re-suspended in M9 buffer (1× BD Difco M9 Salts). Targeted dilutions after cells reached density of OD_600nm_ 0.6 were prepared in M9 buffer and 5 µL aliquots were dispensed in series on an agar plate of M9 selection media containing 0.5 mM diamide, 1 g/L yeast extract, 10 g/L glucose, 0.5% arabinose, 0.5 mM IPTG, ampicillin, kanamycin, chloramphenicol and serial NMN^+^ concentrations of 0, 2, 4 and 6 mM. Selection plates were incubated at 37 °C, and photos were taken to document growth progress.

### Selection of TS-PTDH library

A total of 50 µL pre-made SHuffle/pLZ311 or SHuffle/pLZ312 electro-competent cells were transformed with 2 µL TS-PTDH A155N-E175-A176-L208 NNK library DNA (pLZ316) via electroporation. After rescue in SOC media for 1 h, cultures were combined in 10 mL 2xYT with 10 g/L glucose, 0.5% arabinose, kanamycin and ampicillin. 0.2, or 1 μL of culture was immediately taken from the culture and plated on an 2xYT agar plates with kanamycin and ampicillin. The plate was incubated at 37 °C overnight, and the number of colonies formed was counted to estimate the library size. The liquid cultures were incubated at 37 °C with shaking for 10 h, 1 mL cells were washed 3 times and re-suspended in M9 Buffer. Targeted serial dilutions of cells were prepared in M9 buffer and 5 µL aliquots were dispensed in series on an agar plate of M9 selection media containing 0.5 mM diamide, 5 or 1 g/L yeast extract, 10 g/L glucose, 0.5% arabinose, 0.5 mM IPTG, 10 g/L sodium Pt, kanamycin, ampicillin, and 5 mM NMN^+^. Selection plates were incubated at 37 °C, and photographs were taken to document growth progress. The independent transformants sampled was calculated as 2.4 × 10^6^ and 2.5 × 10^6^ colonies when plated on 5 and 1 g/L yeast extract, respectively. TS-PTDH and the template of the library, TS-PTDH A155N, were cultured in the same conditions as controls. The colonies were numbered by the order of appearance. The selected cells were re-streaked on fresh agar plate with identical media and incubated until single colonies formed on the plate. Variants that cannot form a single colony may be false positives. After extracting the plasmids from each re-streaked colony, the chosen TS-PTDH variants were amplified and moved into the pQElac vector containing a N-terminal 6 × His tag for protein purification and characterization. To demonstrate recapitulation of the growth phenotype, purified TS-PTDH and Gor variant plasmids were transformed identically to the growth selection system. 2 µL of a serial dilution of cells were deposited onto a growth selection plate and grown at 37 °C.

### Purification and characterization of TS-PTDH variants

The *E. coli* BL21(DE3) strains harboring pQElac plasmids encoding the TS-PTDH or TS-PTDH variants were grown in 2xYT media with ampicillin at 37 °C. The IPTG-inducible expression, Ni-affinity purification, and protein concentration determination was conducted as described above.

The specific activities of TS-PTDH and variants were measured with the following methods^64^. Briefly, the reaction mixture contained 100 mM MOPS buffer, pH 7.25, 10 mM sodium Pt, and 4 mM cofactor (NAD^+^, NADP^+^, or NMN^+^). The formation of reduced coenzyme was measured by light absorption at 340 nm in a 96-well plate at 25 °C. Absorption was correlated to a molar basis using the extinction coefficient (ε) of 6.22 mM^−1^ cm^−1^ for each cofactor.

The steady-state kinetic parameters toward the coenzymes were measured by spiking purified protein into a 100 μL reaction mixture containing 100 mM MOPS, pH 7.25, 10 mM sodium Pt, and varied cofactor concentrations from 0.02 mM to 5 mM at 25 °C. The kinetic parameters were measured by monitoring the consumption of reduced coenzyme at 340 nm. Data were fitted to the Michaelis–Menten equation to generate estimates of *K*_*M*_ and *k*_*cat*_ values.

### Molecular modeling

Homology models for mutant TS-PTDH and Ec Gor were generated with Rosetta^65^. Protein structures were visualized with Schrodinger PyMOL software. The crystal structure used as template for TS-PTDH had PDB identifier 4E5N^43^, and the template for Ec Gor was 1GET^66^. Coordinates for NMN/H used in ligand docking were extracted from the co-crystallized nicotinamide cofactor in each template, NAD^+^ for 4E5N and NADP^+^ for 1GET, and the remaining AMP atoms were deleted. The Rosetta docking protocol involved mutation from the WT structure, repeated rounds of random rigid-body perturbation by translation and rotation for NMN/H, and optimization of active site rotamers through side chain repacking and minimization^67^. All moves were sampled with the Monte Carlo method, coordinate restraints were utilized to maintain the NMN/H in a catalytically competent pose, and full flexibility for ligand and protein backbone torsions was allowed to identify the optimal binding pose. A total of 1000 docking trials was run for each variant, the top 100 models based on total Rosetta energy (an aggregate of residue energy terms describing van der Waals, electrostatics, rotamer conformation probabilities, etc. that serves as an indicator of complex stability) were sorted on interface energy scores (the difference in energy with the ligand separated from the complex that reflects the predicted favorability of ligand binding) and the model with the most favorable interface energy to NMN/H was selected as the reference following visual inspection to ensure no structural artifacts^68^.

### TS-PTDH total turnover number determination

Reactions to determine the TTN of TS-PTDH LY-13 (Fig. 4B) were performed at 1 mL volumes in 2 mL glass vials secured with a PTFE-lined screw cap. Reactions were incubated without shaking at 30 °C. Reaction buffer was comprised of 200 mM MOPS at pH 7.5, 100 mM sodium Pt, 6 mM NMN^+^, and 33 mM KIP. To initiate the reaction, purified TS-PTDH LY-13 and XenA were spiked into the reaction mixture to a final concentration of 0.25 µM and 18.8 µM, respectively. The reactions were sampled intermittently by removing 50 µL of reaction mixture and mixing with 100 µL of ethyl acetate containing 200 mg/L 1-octanol. After extraction, the ethyl acetate fraction was transferred to a GC vial for GC-FID analysis. GC-FID analysis for KIP biotransformation efficiency was performed on an Agilent 6850 GD using an Agilent DB-WAX column (30 m × 0.56 mm × 1 µm). Helium was used as a carrier gas. The inlet and detector were held at 250 and 260 °C, respectively. 5 µL of sample was injected with a 2:1 split ratio. The oven was held at 200 °C for 15 min. TS-PTDH LY-13 was added at a low concentration to require many turnovers of LY-13 for the reaction to progress. TTN was calculated by the moles of levodione produced divided by the moles of LY-13 supplied to the reaction system. Total turnover frequency was determined by dividing TTN by the total reaction time of 384 h. Levodione concentration was determined by correlating sample response to a standard curve of known values.

### Comparison of PTDH and GDH Triple redox cofactor cycling systems

In cycling reactions focused on higher conversion and productivity, Fig. 4C, the concentration of cofactor cycling protein (TS-PTDH or GDH Triple) was increased relative to the TTN determination experiments. Reactions were performed at 30 °C without shaking in 1 mL volumes in 2 mL glass vials secured with a PTFE-lined screw cap. Sampling and GC-FID analysis were performed as described in the TTN method section.

Reactions utilizing TS-PTDH LY-13 contained 100 mM MOPS at pH 7.5, 100 mM sodium Pt, 33 mM KIP, and 18.8 µM XenA. Reactions utilizing GDH Triple contained 200 mM potassium phosphate at pH 7.5, 300 mM D-glucose, 1 M NaCl, 33 mM KIP, and 18.8 µM XenA^16^. The NMN^+^ concentration in each reaction was varied at 0, 0.05, 0.1, 0.5, or 5 mM. The reactions were initiated by spiking TS-PTDH LY-13 or GDH Triple to a final concentration of 10 µM.

### BNA(H)-mediated PTDH biotransformation

Reactions with BNA^+^ (Fig. 4D) contained 100 mM MOPS-NaOH at pH 7.6, 10 mM KIP, 100 mM sodium Pt, and 2% v/v DMSO. BNA^+^Cl^-^, prepared by refluxing equimolar amounts of nicotinamide and benzyl chloride in acetonitrile overnight, followed by precipitation with diethyl ether on ice^10^, was supplied to the reactions at final concentrations of 0, 5, 10, or 20 mM. Reactions were initiated by spiking *Ts*OYE and the appropriate TS-PTDH variant to a final concentration of 10 µM, each. Reaction mixtures were incubated at 30 °C on a Thermomixer C (Eppendorf) with shaking at 900 rpm for 24 h, extracted with 500 µL ethyl acetate containing 5 mM *n*-dodecane, and analyzed by GC-FID. The cycling system with no BNA^+^ was used to determine the background activity of the cycling systems. This value was subtracted from the final levodione concentrations in the 5, 10, and 20 mM samples to determine the BNA(H)-specific levodione production. Concentrations of levodione product were determined using a calibration line (0–10 mM, 6 levels, using *n*-dodecane as internal standard). The y-intercept of the calibration line was forced through 0.

For BNA(H)-mediated reactions, gas chromatography analyses were performed on a GC-2010 (Shimadzu, Japan) equipped with an AOC-20i auto injector and a flame ionization detector (FID), using a CP-Sil 5 column (25 m × 0. 25 mm × 1.2 µM). 1 µL of sample was injected with a split ratio of 100:1 and injector at 340 °C. The FID was maintained at 360 °C. Nitrogen was used as the carrier gas, with a linear velocity of 30 cm/s. The oven was held at 135 °C for 1 min, ramped at 15 °C/min to 215 °C, then 30 °C/min to 345 °C, and held at 345 °C for 1 min.

### Expression and purification of proteins for BNA(H)-mediated biotransformation reactions

*E. coli* BL21(DE3) strains were transformed with the plasmids containing the TS-PTDH variants and grown on LB-agar plates containing ampicillin at 37 °C overnight. An overnight culture, 15 mL LB supplemented with ampicillin, was inoculated with a single colony, and grown at 37 °C, 170 rpm overnight. 2 L baffled shake-flasks with 250 mL of 2x YT media containing 200 µg/mL ampicillin, were inoculated with the overnight culture, grown at 37 °C, 170 rpm until OD_600_ of ~1.0, induced with IPTG (0.5 mM), and grown at 30 °C and 170 rpm for 24 h. Cells were harvested by centrifugation at 18,000 × *g*, 4 °C, 10 min and stored at −20 °C until purification.

The pellet was resuspended in four volumes loading buffer (50 mM sodium phosphate, 300 mM sodium chloride, 10 mM imidazole, pH 7.7), and lysed on ice by sonication (Branson sonifier 250; 15 min total, 40% duty cycle, output control 3.5, 2 mm tip). The lysate was clarified (21,000 × *g*, 4 °C, 30 min followed by 0.2 µm filtration), and loaded (1 mL/min) onto a Nickel-HisTrap FF crude (1 mL) column, washed with loading buffer (12 column volumes, CV), followed by a step with 10% elution buffer (loading buffer containing 250 mM imidazole; 12 CV). The protein was then eluted with 100% elution buffer and collected in fractions of 0.5 mL. Fractions containing protein were combined and dialyzed against ice-cold dialysis buffer (50 mM sodium phosphate, pH 7.7, 800 mL); the buffer was renewed after the first 2 h of dialysis. Following the dialysis, the protein was mixed with glycerol (20% *v/v* final concentration). Enzyme concentrations were estimated by the absorbance at 280 nm (non-denatured protein), using predicted extinction coefficients (wild type and LY-13: 26470, LY-7: 31970 mM^−1^ cm^−1^; https://web.expasy.org/protparam/). The protein was aliquoted, flash frozen in liquid nitrogen, and stored at −80 °C until use.

The *Ts*OYE was produced and purified identically to methods previously described^69^. Briefly, *E. coli* BL21(DE3) strains harboring the gene for *Ts*OYE (grown at 37 °C, 180 rpm) were induced at OD_600_ of ~0.6 with IPTG (0.1 mM, 30 min, 4 °C), resuspended in MOPS-NaOH (20 mM, pH 7.0), disrupted using a Multi Shot Cell Disruption System (two cycles) and clarified by centrifugation (17,500 × *g*, 30 min, 4 °C). The supernatant was incubated at 70 °C for 90 min, clarified by centrifugation (38,500 × *g*, 30 min, 4 °C), supplemented with FMN (1:1 molar ratio), incubated on ice (30 min), washed and concentrated with MOPS-NaOH (20 mM, pH 7.0) using an AMICON filter with 30 kDa cut-off. Purity was assessed by SDS-PAGE and mass spectrometry, and the enzyme was stored as aliquots at −20 °C until use.

### Reporting summary

Further information on research design is available in the Nature Research Reporting Summary linked to this article.

## Supplementary information

Supplementary Information

Description of Additional Supplementary Files

Supplementary Data 1

Supplementary Data 2

Reporting Summary

## Data Availability

Data supporting the findings of this work are available within the paper and its Supplementary Information files. A reporting summary for this paper is available as a Supplementary Information file. Source data are provided with this paper.

## Source data

Source Data

## Code availability

Source code are available at GitHub [https://github.com/hanli-lab/Gor-PTDH].
