# Supplementary material for: Directed evolution of phosphite dehydrogenase to cycle noncanonical redox cofactors via universal growth selection platform
Source: Nat Commun. 2022 Aug 26;13:5021. doi: 10.1038/s41467-022-32727-w (PMC9418148; doi:10.1038/s41467-022-32727-w)
Supplement: Supplementary file 1 — Supplementary Information [file 41467_2022_32727_MOESM1_ESM.pdf]

Directed evolution of phosphite dehydrogenase to cycle noncanonical redox cofactors via universal growth selection platform

Zhang and King *et al*.

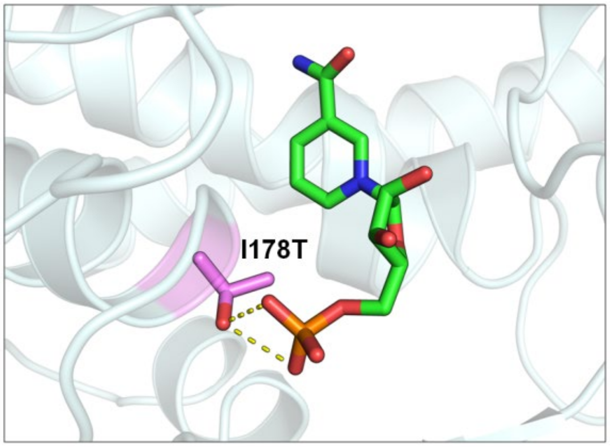

Supplementary Figure 1. Model of NMN^+ binding pose with Ec Gor I178T. The hydrophobic I178 is unable to make attractive polar contacts with NMN^+. With mutation to I178T, the threonine is positioned to form hydrogen bonds with the NMN^+ phosphate group.

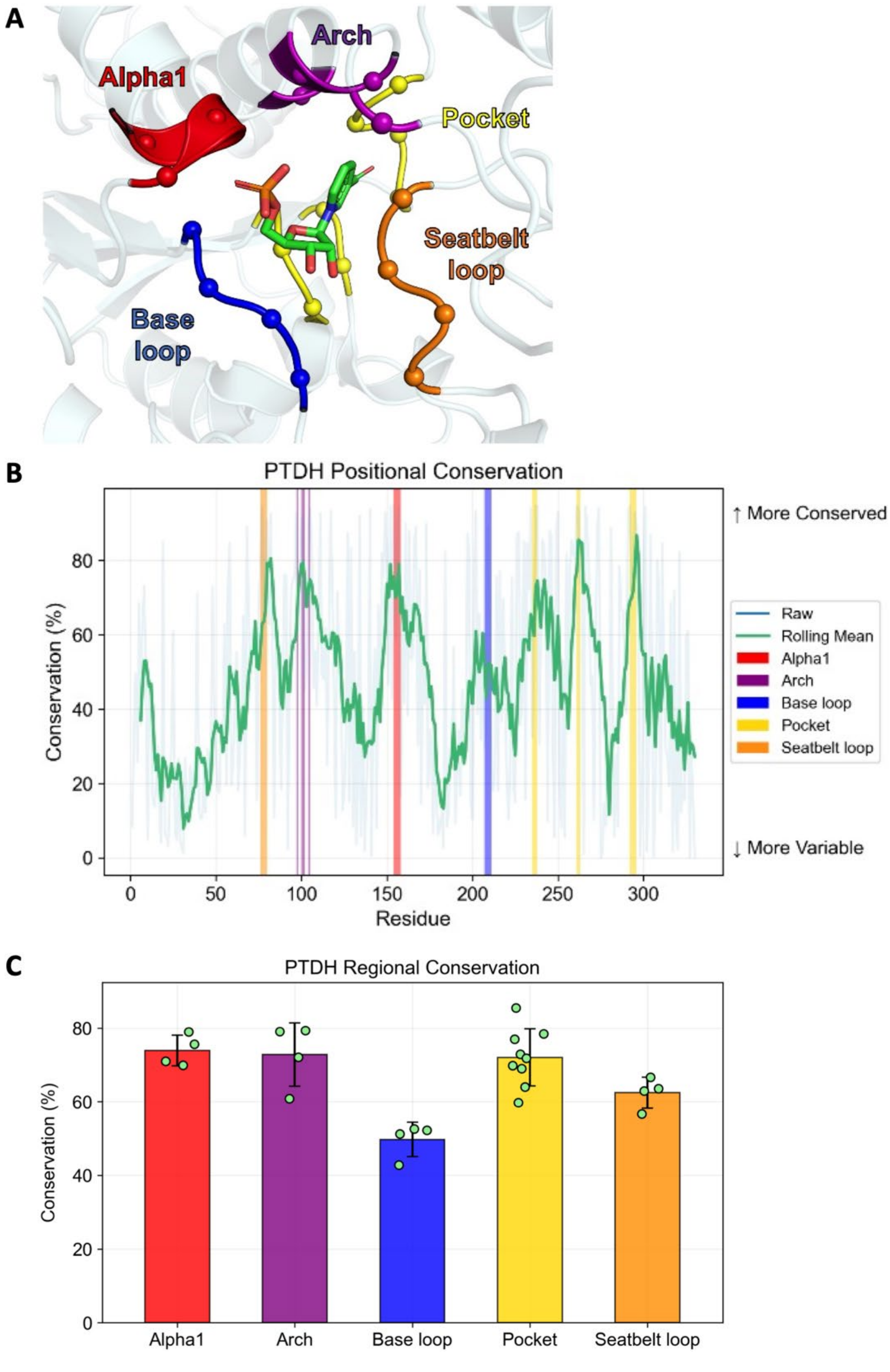

Supplementary Figure 2. TS-PTDH active site bioinformatic analysis. A. First-shell binding residues surrounding NMN^+ were identified as positions containing any atom within 6 Å of the predicted NMN^+ binding pose. Since NMN^+ and NAD^+ share the same core structure with hydride transfer occurring at the nicotinamide ring C4, we hypothesize that the active binding mode will be almost identical. Five regions were selected for conservation analysis: 1) the seatbelt loop from 76-79 named for the characteristic K76 found in many PTDHs that reaches across the binding pocket to form a polar contact with the pyrophosphates[1], 2) the arch above which includes 97,100, 101, and 104, 3) the Rossmann alpha helix 1 from 154-157, 4) the base loop running from 207-210, and 5) the nicotinamide pocket which includes 235-

237, 261-262, and 292-295. Numbered residues are illustrated in Supplementary Figure 2A. B. Positional conservation of PTDH residues. The raw signal is illustrated as light blue line, and a smoothed signal with 6-window moving average is shown in green line to focus on regional variation. The conservation score is a relative ranking with higher values indicating that the position has greater conservation, and lower values meaning greater variance. C. Mean conservation scores for the regions composing the NMN^+ binding site. Residues belonging to the specified region are represented as a green dot, and the standard deviation of the conservation scores is shown as error bars, there are no replicates as this is an aggregate of the individual positions. All regions show a high degree of conservation with values ranging from 50 ± 4 % for the base loop to 74 ± 4 % for the Rossmann alpha helix 1. Mutations in these areas would strongly affect NAD^+ turnover due to their direct contact and variants with reduced catalytic efficiency for NAD^+ would suffer a considerable loss in fitness. As usage of mNADs is not found naturally, there is little selection pressure to sample sequences away from those already proficient with NAD^+. The bioinformatic analysis of the cofactor active site is ineffective in discovering evolvable, variable regions that readily tolerate mutations for mNAD engineering. Analysis was performed as described in Supplementary Method 1. Source data are provided as a Source Data file.

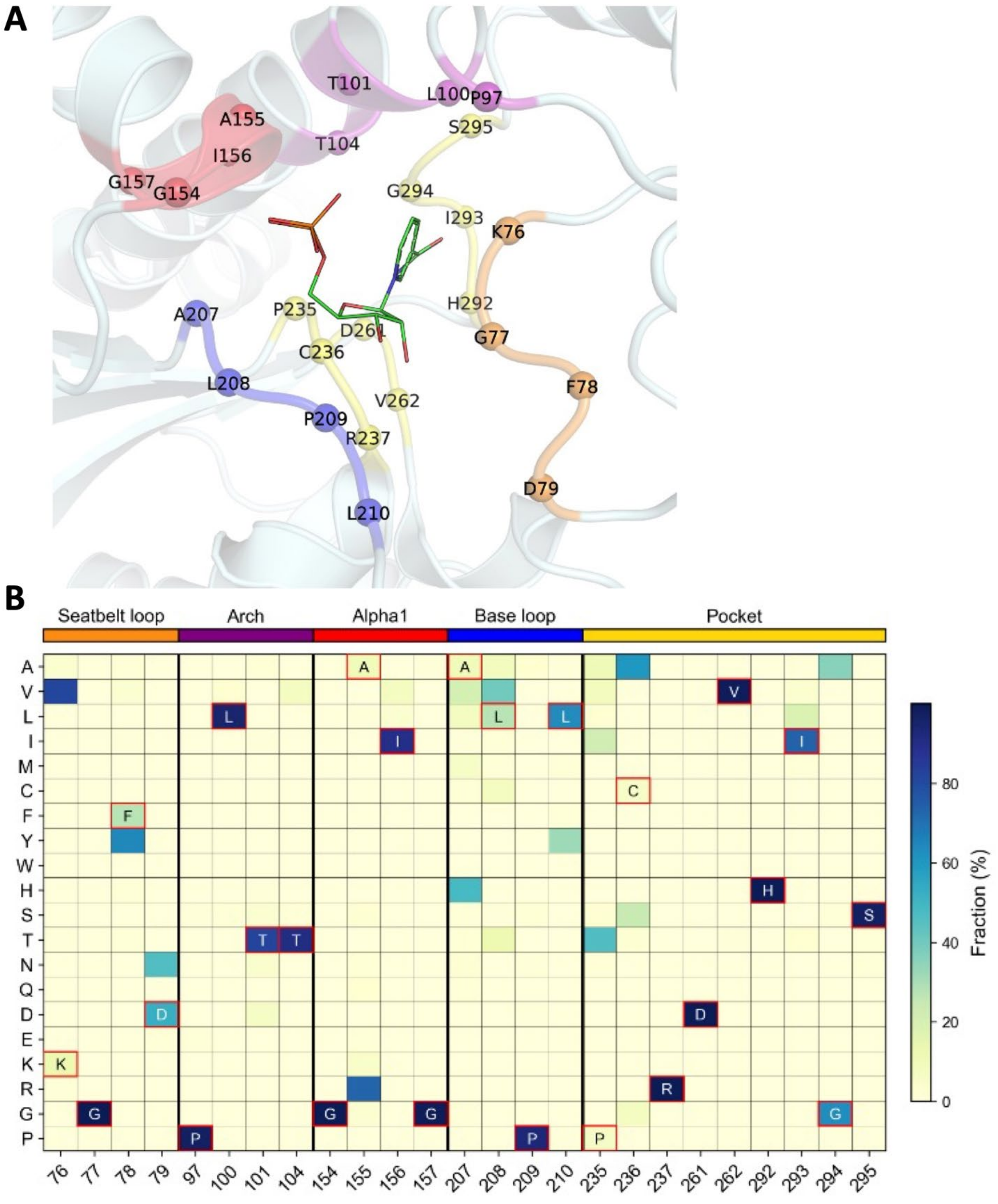

Supplementary Figure 3. TS-PTDH active site residue frequencies. A. First shell residues enveloping the NMN$^+$ (green lines) are colored according to their regions illustrated in Figure S1A, each residue alpha carbon is represented as a sphere and labeled with the amino acid identity and position. B. The distribution of residue frequencies with gaps ignored for each position was calculated from multiple sequence alignment

(MSA) of TS-PTDH homologs found from BLAST-P search and colored by frequency with yellow indicating 0% observation to dark blue indicating 100% observation. The wild-type residue at each position is highlighted with red outline and text annotation. This analysis was performed to detect if any position was amenable to sampling polar residues that are arranged to potentially form favorable electrostatic contact with the NMN^+ phosphate and to visualize the types of residues natively found. The existence of any position with recurrent substitution to polar residues would give strong evidence that the mutations would be tolerated on the TS-PTDH scaffold and additionally the sequences with the substitutions could be an alternative template for engineering. The results exhibit severe purifying selection as all regions are highly conserved and the small amount of variability observed is generally limited to positions swapping between small polar or non-polar amino acids. Due to the lack of suggested variants from evolutionary analysis, we focus on applying structure-based rational design to engineer NMN^+ binding.

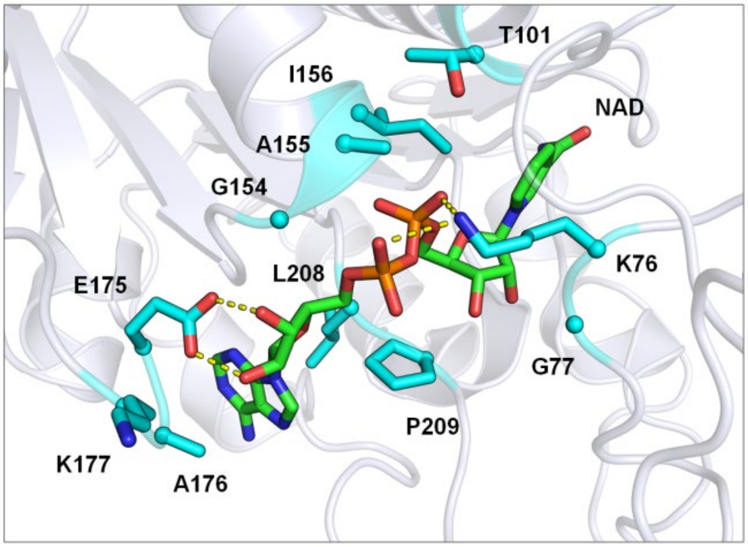

Supplementary Figure 4. Binding mode of the natural cofactor NAD^+ in TS-PTDH (PDB: 4E5N). First shell residues lining the cofactor binding pocket are indicated by cyan sticks. Critical side-chain polar contacts that secure NAD^+ include K76 which reaches from a loop across the binding interface to form salt bridges with the pyrophosphate, and E175 that establishes a bidentate hydrogen bond with the adenosine ribose.

According to the crystal structure of TS-PTDH bound with NAD^+, there are 11 residues lining the NAD^+ binding pocket that can be potentially designed: K76, G77, T101, G154, A155, 1156, E175, A176, K177, L208, and P209. We first excluded the positions E175, K177 and A176 as they are distal from the NMN^+ phosphate and single mutations at these positions cannot form direct interactions with NMN^+. We also dismissed position K76 which establishes a salt-bridge with the pyrophosphate in NAD^+ and NMN^+ phosphate. This side-chain polar contact is critical, validated by the variant K76R that completely lacks NMN^+ activity (Figure 2A). Taken together, 6 positions (T101, A155, G154, L208, G77, and P209) were selected for the design efforts. These designable positions were mutated to polar amino acids, with preference for positively charged residues to form novel polar contacts with NMN^+. The resulting variants were evaluated based on their shape complementarity and the possibility to form new hydrogen bonds through visual inspection. Overall, 14 variants were selected and characterized for NMN^+ activity (Figure 2A).

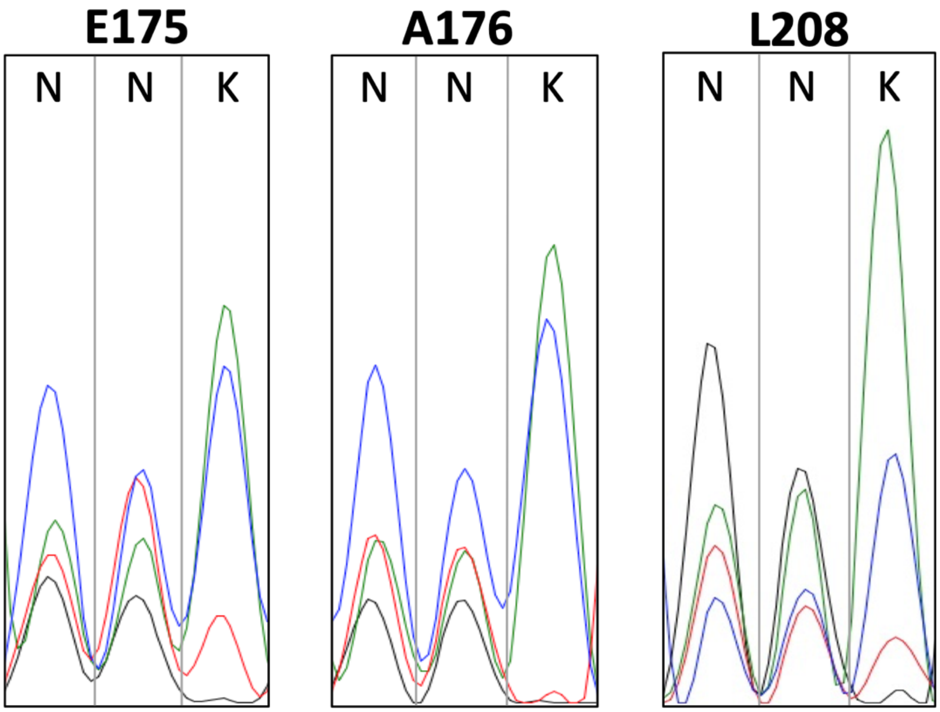

E175

	N	N	K
T	0.236	0.226	0.479
A	0.192	0.308	0.107
C	0.160	0.145	0.011
G	0.412	0.321	0.403
$\sum_{dev}$	0.675	0.743	1.000
Q_{pool}	0.834		

A176

	N	N	K
T	0.215	0.237	0.541
A	0.217	0.239	0.009
C	0.131	0.159	0.000
G	0.437	0.365	0.451
$\sum_{dev}$	0.627	0.770	0.919
Q_{pool}	0.816		

L208

	N	N	K
T	0.241	0.322	0.632
A	0.191	0.146	0.280
C	0.438	0.177	0.075
G	0.130	0.355	0.014
$\sum_{dev}$	0.623	0.647	0.736
Q_{pool}	0.754		

Supplementary Figure 5. Analysis of library quality at site saturated mutagenesis targeted residues. Sanger sequencing of the TS-PTDH library used for growth selection shows a distribution of bases at each NNK site. Library quality was determined as described by Stewart and coworkers[2,3]. Briefly, peak heights of the fluorescent chromatogram from Sanger Sequencing were determined at each nucleotide position, summed, and standardized by the summed height at that base, as shown in the tables below each chromatogram. Relative height values were then used to determine the summed amount of deviation from the expected distribution, $\sum_{dev}$. For example, an "N" basepair should show an even 25% relative height of each of the four bases, where a "K" site should show only a 50% relative distribution between "T" and "G". Finally, the Q_{pool} value is determined as a weighted metric of each base in the codon. Q_{pool} values > 0.7 are regarded as satisfactory for library quality[2,3].

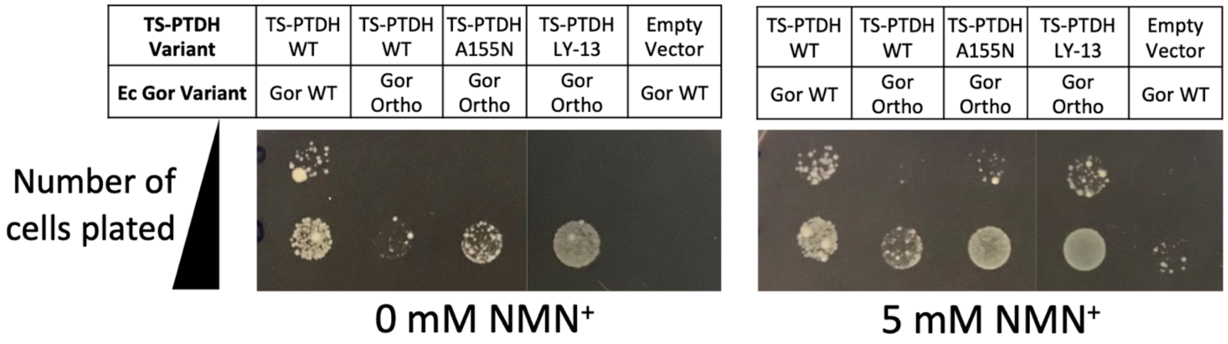

Supplementary Figure 6. Recapitulation of growth restoration phenotype for *E. coli* Shuffle strain with wild-type TS-PTDH, variants from rational design, and variants identified in selection spotted onto agar plates. Co-expression of wild type TS-PTDH and wild type Ec Gor supported cell growth, while cells with co-expression of wild type TS-PTDH and NMNH-specific Gor Ortho grew poorly. When TS-PTDH mutants were introduced, co-expression of TS-PTDH A155N with NMNH-specific Gor Ortho restored growth at a low level, but co-expression of selected TS-PTDH LY-13 (A155N-E175A-A176F) with NMNH-specific Gor Ortho enabled improved growth compared to TS-PTDH A155N. Co-expression of an empty plasmid and wild-type Gor struggled to grow in the same conditions.

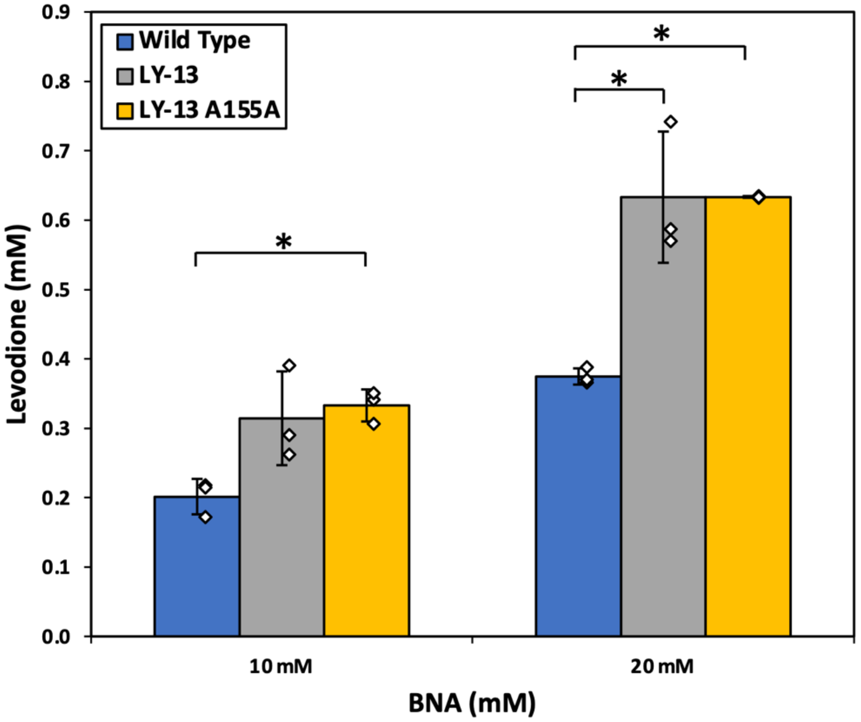

Supplementary Figure 7. Reversion of the A155 mutation in LY-13 retains activity with BNA^+. The A155N mutation in LY-13, the highest performing TS-PTDH variant with BNA^+, was reverted to an alanine. When LY-13 A155A was cycled with *Ts*OYE and BNA^+, biotransformation activity remained consistent with LY-13. Reactions were performed as described in the "BNA(H)-Mediated PTDH Biotransformation" methods section. Values represent the average of three biological replicated. Error bars represent one standard deviation. Statistical significance was determined by two-tailed t-test, (* $P<0.05$). For statistics, Wild Type vs LY-13 A155A at 10 mM BNA^+, P = 0.002; Wild Type vs LY-13 at 20 mM BNA^+, P = 0.009; Wild Type vs LY-13 A155A at 20 mM BNA^+, P = 2 x 10^{-6}. Source data are provided as a Source Data file.

Supplementary Table 1. Kinetic parameters of Gor variants.

Enzyme	Kinetic parameters								
	NADH			NADPH			NMNH		
	k_{cat}[s^{-1}]	K_m[mM]	k_{cat} / K_m [mM^{-1} s^{-1}]	k_{cat}[s^{-1}]	K_m[mM]	k_{cat} / K_m [mM^{-1} s^{-1}]	k_{cat}[s^{-1}]	K_m[mM]	k_{cat} / K_m [mM^{-1} s^{-1}]
Gor	n.d.*	n.d.*	0.04 ± 0.01	84±7	0.06 ± 0.01	1450 ± 119	n.d.*	n.d.*	0.003 ± 0.001
Gor I178T	n.d.*	n.d.*	0.09 ± 0.01	31±1	0.04 ± 0.01	874 ± 160	n.d.*	n.d.*	0.014 ± 0.003
Gor I178T-R198M-R204L (Gor Ortho)	n.d.*	n.d.*	0.06 ± 0.01	n.d.*	n.d.*	0.10 ± 0.01	n.d.*	n.d.*	0.012 ± 0.001

Reactions were performed in 96 mM Tris-Cl buffer pH 7.5, 2.5 mM GSSG and varied cofactor concentrations. Values reported with standard deviation and a replicate size of n = 3. Source data are provided as a Source Data file.

* Not detectable. K_m values are higher than 1.5 mM as the enzyme could not be saturated with the cofactor concentrations tested. For k_{cat}/K_m, the Michaelis-Menten equation was modified under the assumption $K_m >> S$, as previously described[1]. Using the modified equation, we performed linear regression on initial reaction rate versus NMNH concentration, the k_{cat}/K_m was determined using the resulting slope divided by enzyme concentration.

Supplementary Method 1. TS-PTDH binding site conservation analysis

Sequence conservation was analyzed by performing a BLAST-P[4] search using 5,000 max target sequences with TS-PTDH as the query to identify homologs. Hits were filtered to remove samples with low query coverage (<60%), low sequence identity (<30%), and redundant sequences (>98% sequence identity). Multiple sequence alignment with MAFFT[5] was run, and frequencies for each of the 20 amino acids were tabulated for every non-gap column based on TS-PTDH. Sequence entropy was calculated using ProDy[6] at each position with the formula where H is entropy in bits, p_i is the observed frequency for an amino acid, and the values are summed over the 20 natural residues with gaps ignored.

$$H(X) = -\sum_{i=1}^{20} p_i \log_2 p_i \quad \textbf{(1)}$$

Sequence entropy was converted to z-score and percentile rank. A final conservation score was computed by subtracting the sequence entropy percentile from 1 to rank how conserved each residue is compared to the entire population with a score of 0 indicating max variability and 1 indicating max conservation.

Five regions of interest composing the first shell of residues surrounding the NMN^+ were examined based on the crystal structure of TS-PTDH with NAD^+ bound (PDB: 4E5N)[7]. The catalytically active NMN^+ binding pose is assumed to be similar to that of the native cofactor NAD^+ as NMN^+ fully maintains the nicotinamide ring involved in hydride transfer. These regions are: 1) the seatbelt loop from 76-79, 2) the arch which includes 97, 100, 101, and 104, 3) the Rossmann alpha helix 1 from 154-157, 4) the base loop with 207-210, and 5) the nicotinamide pocket which includes 235-237, 261-262, and 292-295.

Supplementary references

1. Woodyer, R. et al. Site-directed mutagenesis of active site residues of phosphite dehydrogenase. *Biochemistry*. **44**, 4765-4774 (2005).
2. Sullivan, B., Walton, A. Z. & Stewart, J. D. Library construction and evaluation for site saturation mutagenesis. *Enzyme Microb. Technol.* **53**, 70-77 (2013).
3. Walton, A. Z., Sullivan, B., Patterson-Orazem, A. C. & Stewart, J. D. Residues controlling facial selectivity in alkene reductase and semirational alterations to create stereocomplementary variants. *ACS Catal.* **4**, 2307-2318 (2014).
4. Camacho, C. et al. BLAST+: architecture and applications. *BMC Bioinf.* **10**, No. 421 (2009).
5. Katoh, K., Misawa, K., Kuma, K. & Miyata, T. MAFFT: a novel method for rapid multiple sequence alignment based on fast Fourier transform. *Nucleic Acids Res.* **30**, 3059-3066 (2002).
6. Bakan, A. Meireles, L. M. & Bahar, I. *ProDy*: protein dynamics inferred from theory and experiments. *Bioinformatics*. **27**, 1575-1577 (2011).
7. Zou, Y. et al. Crystal structures of phosphite dehydrogenase provide insights into nicotinamide cofactor regeneration. *Biochemistry*. **51**, 4263-4270 (2012).
