# Supplementary material for: Directed evolution of phosphite dehydrogenase to cycle noncanonical redox cofactors via universal growth selection platform
Source: Nat Commun. 2022 Aug 26;13:5021. doi: 10.1038/s41467-022-32727-w (PMC9418148; doi:10.1038/s41467-022-32727-w)
Supplement: Supplementary file 2 — Description of Additional Supplementary Files [file 41467_2022_32727_MOESM2_ESM.pdf]

File Name: Supplementary Data 1
Description: List of strains and plasmids used in this study.

File Name: Supplementary Data 2
Description: Accession codes for crystal structures and genes used in this study.
