# Supplementary material for: Directed evolution of phosphite dehydrogenase to cycle noncanonical redox cofactors via universal growth selection platform
Source: Nat Commun. 2022 Aug 26;13:5021. doi: 10.1038/s41467-022-32727-w (PMC9418148; doi:10.1038/s41467-022-32727-w)
Supplement: Supplementary file 5 — Reporting Summary [file 41467_2022_32727_MOESM5_ESM.pdf]

nature portfolio

Corresponding author(s): Han Li

Last updated by author(s): Aug 2, 2022

Reporting Summary

Nature Portfolio wishes to improve the reproducibility of the work that we publish. This form provides structure for consistency and transparency in reporting. For further information on Nature Portfolio policies, see our Editorial Policies and the Editorial Policy Checklist.

Statistics

For all statistical analyses, confirm that the following items are present in the figure legend, table legend, main text, or Methods section.

n/a	Confirmed	
☐	☒	The exact sample size (*n*) for each experimental group/condition, given as a discrete number and unit of measurement
☐	☒	A statement on whether measurements were taken from distinct samples or whether the same sample was measured repeatedly
☐	☒	The statistical test(s) used AND whether they are one- or two-sided<br>*Only common tests should be described solely by name; describe more complex techniques in the Methods section.*
☒	☐	A description of all covariates tested
☐	☒	A description of any assumptions or corrections, such as tests of normality and adjustment for multiple comparisons
☐	☒	A full description of the statistical parameters including central tendency (e.g. means) or other basic estimates (e.g. regression coefficient) AND variation (e.g. standard deviation) or associated estimates of uncertainty (e.g. confidence intervals)
☐	☒	For null hypothesis testing, the test statistic (e.g. *F*, *t*, *r*) with confidence intervals, effect sizes, degrees of freedom and *P* value noted<br>*Give P values as exact values whenever suitable.*
☒	☐	For Bayesian analysis, information on the choice of priors and Markov chain Monte Carlo settings
☒	☐	For hierarchical and complex designs, identification of the appropriate level for tests and full reporting of outcomes
☒	☐	Estimates of effect sizes (e.g. Cohen's *d*, Pearson's *r*), indicating how they were calculated

Our web collection on statistics for biologists contains articles on many of the points above.

Software and code

Policy information about availability of computer code

Data collection	GC data were collected using ChemStation B.04.03.<br>Enzyme assay and cell growth data were collected using SoftMax Pro 7.0.<br>Enzyme modeling was performed with PyRosetta 2018.52+release.674a9609eaf and Rosetta 2018.48.60516.<br>Protein structures were visualized with Schrodinger PyMOL 2.3.4.
Data analysis	Data analysis was performed with python 3.7, numpy 1.19.1, pandas 0.24.2, and Microsoft Excel 16.

For manuscripts utilizing custom algorithms or software that are central to the research but not yet described in published literature, software must be made available to editors and reviewers. We strongly encourage code deposition in a community repository (e.g. GitHub). See the Nature Portfolio guidelines for submitting code & software for further information.

Data

Policy information about availability of data

All manuscripts must include a data availability statement. This statement should provide the following information, where applicable:

- Accession codes, unique identifiers, or web links for publicly available datasets
- A description of any restrictions on data availability
- For clinical datasets or third party data, please ensure that the statement adheres to our policy

The authors declare that all relevant data supporting these findings are available in the main text, supplementary data, and supplementary information. Accession

codes are available in the Supplementary Data. Custom code used in this study is available on GitHub[https://github.com/hanli-lab/Gor-PTDH].

Human research participants

Policy information about studies involving human research participants and Sex and Gender in Research.

Reporting on sex and gender	n/a
Population characteristics	n/a
Recruitment	n/a
Ethics oversight	n/a

Note that full information on the approval of the study protocol must also be provided in the manuscript.

Field-specific reporting

Please select the one below that is the best fit for your research. If you are not sure, read the appropriate sections before making your selection.

[x] Life sciences [ ] Behavioural & social sciences [ ] Ecological, evolutionary & environmental sciences

For a reference copy of the document with all sections, see nature.com/documents/nr-reporting-summary-flat.pdf

Life sciences study design

All studies must disclose on these points even when the disclosure is negative.

Sample size	No specific calculations were used to determine sample size for each experiment. All experiments were performed with sample sizes of n = 3 unless otherwise state. Sample sizes of n=2 were used in screening initial protein mutant candidates, and a third replicate was performed with strong positive activity hits. Selected sample sizes were consistent with previous comparable studies in the field (Johannes et al. Biotechnology and Bioengineering, 98, 2006. Woodyer et al. FEBS, 272, 2005. Beyer et al. Appl Microbiol Biotechnol, 101, 2017.)
Data exclusions	No data were excluded from analyses.
Replication	All replication attempts were successful. Each time experiments were performed, controls of known activity levels (positive and negative) accompanied the unknown samples, and the controls were in agreement with previous experiments. All experiments were independent.
Randomization	All samples, cultures, and replicates were treated identically. Therefore, randomization was not relevant.
Blinding	This study reports objective measurements of samples derived from bacterial culture, purified proteins, or chemical reactions. In each experiment, the samples were treated identically. Therefore, blinding was not relevant.

Reporting for specific materials, systems and methods

We require information from authors about some types of materials, experimental systems and methods used in many studies. Here, indicate whether each material, system or method listed is relevant to your study. If you are not sure if a list item applies to your research, read the appropriate section before selecting a response.

Materials & experimental systems

n/a	Involved in the study	
[x]	[ ]	Antibodies
[x]	[ ]	Eukaryotic cell lines
[x]	[ ]	Palaeontology and archaeology
[x]	[ ]	Animals and other organisms
[x]	[ ]	Clinical data
[x]	[ ]	Dual use research of concern

Methods

n/a	Involved in the study	
[x]	[ ]	ChIP-seq
[x]	[ ]	Flow cytometry
[x]	[ ]	MRI-based neuroimaging
